# Malignant Cutaneous Neurocristic Hamartoma With Features of Melanoma: A Rare Entity

**DOI:** 10.7759/cureus.69594

**Published:** 2024-09-17

**Authors:** Muhammad Tahir, Abdulhadi Samman, Sara Shalin, Kurt Knowles, Guillermo A Herrera, Joe S Liles, Thuy Phung

**Affiliations:** 1 Pathology and Laboratory Medicine, University of South Alabama College of Medicine, Mobile, USA; 2 Basic Medical Sciences, Pathology Division, College of Medicine, University of Jeddah, Jeddah, SAU; 3 Pathology, University of Arkansas for Medical Sciences, Arkansas, USA; 4 Pathology, University of South Alabama College of Medicine, Mobile, USA; 5 Surgery, University of South Alabama Health System, Mobile, USA

**Keywords:** malignant cutaneous neurocristic hamartomas, malignant neurocristic hamartomas, melanocytic lesion, neurocristic cutaneous hamartoma, neurocristic hamartoma

## Abstract

Neurocristic cutaneous hamartoma (NCH) is a rare, benign neoplastic skin lesion characterized by a combination of neuroectodermal and mesodermal components. Clinically, NCH typically presents as asymptomatic, well-circumscribed, and elevated cutaneous nodules. Histopathologically, it is characterized by nests of pigmented melanocytes and varying degrees of fibrosis and collagen deposition. The precise etiology of NCH remains undetermined; however, it is hypothesized to arise from the aberrant development of neuromesenchyme. Due to its potential to mimic other pigmented melanocytic disorders, accurate differential diagnosis is crucial to prevent mismanagement. Surgical excision is the preferred treatment modality, offering a generally favorable prognosis and low recurrence rate. Conversely, malignant cutaneous neurocristic hamartoma (MCNH), an exceedingly rare malignant variant of NCH, poses a significantly different clinical challenge. This review focuses on the diagnostic criteria, clinical presentation, and management strategies for MCNH, emphasizing the need for differentiation from other similar cutaneous lesions. We present a detailed case report of MCNH in a 56-year-old female, highlighting its histopathological and immunohistochemical features to provide insights into the diagnosis and therapeutic approach for this exceptionally rare malignancy.

## Introduction

Neurocristic cutaneous hamartoma (NCH) is derived from the aberrant development of the neuromesenchyme. This entity was first described by Tuthill et al. in 1982 and referred to as pilar neurocristic hamartoma [1]. NCH of the scalp is a rare entity, and malignant transformation of this benign tumor is extremely rare. According to knowledge and literature research, very few cases of NCH of the scalp have been reported in the English literature [2]. NCH commonly appears as pigmented skin, scalp, and superficial soft tissue lesions composed of Schwann cells, pigmented spindle cells, dendritic cells, and scattered melanocytes. Histologically, NCH is characterized by a proliferation of melanocytes and spindle-shaped Schwann cells in the dermis, often forming nests or clusters. The lesion may include neural elements such as nerve fibers or ganglion cells and displays pigmented dendritic cells positive for S-100 protein. The stroma is typically dense and fibrous, with no significant cytologic atypia or mitotic activity, helping to distinguish it from malignant neoplasms [3]. These lesions can be congenital or acquired and often appear as a blue plaque or patch that increases in size and complexity over time [4]. Malignant transformation of NCH can rarely occur, and there has been only one research paper that discusses the seven cases of malignant NCH published by Pearson et al. [5].

## Case presentation

A 56-year-old Caucasian female with an extensive past medical history of seizures, migraines, depression, anxiety, peripheral neuropathy, hyperlipidemia, hypercholesterolemia, arthritis, type 2 diabetes mellitus, and obesity, presented with a chief complaint of a 1 to 2 cm pink nodule with irregular borders located on the right superior central forehead. Per the patient’s report, the lesion had been present for months and had recently changed in color. Clinical differential diagnosis of benign versus malignant melanocytic lesions was considered and a biopsy of the lesion was done for proper histological diagnosis and treatment.

**Figure 1:**
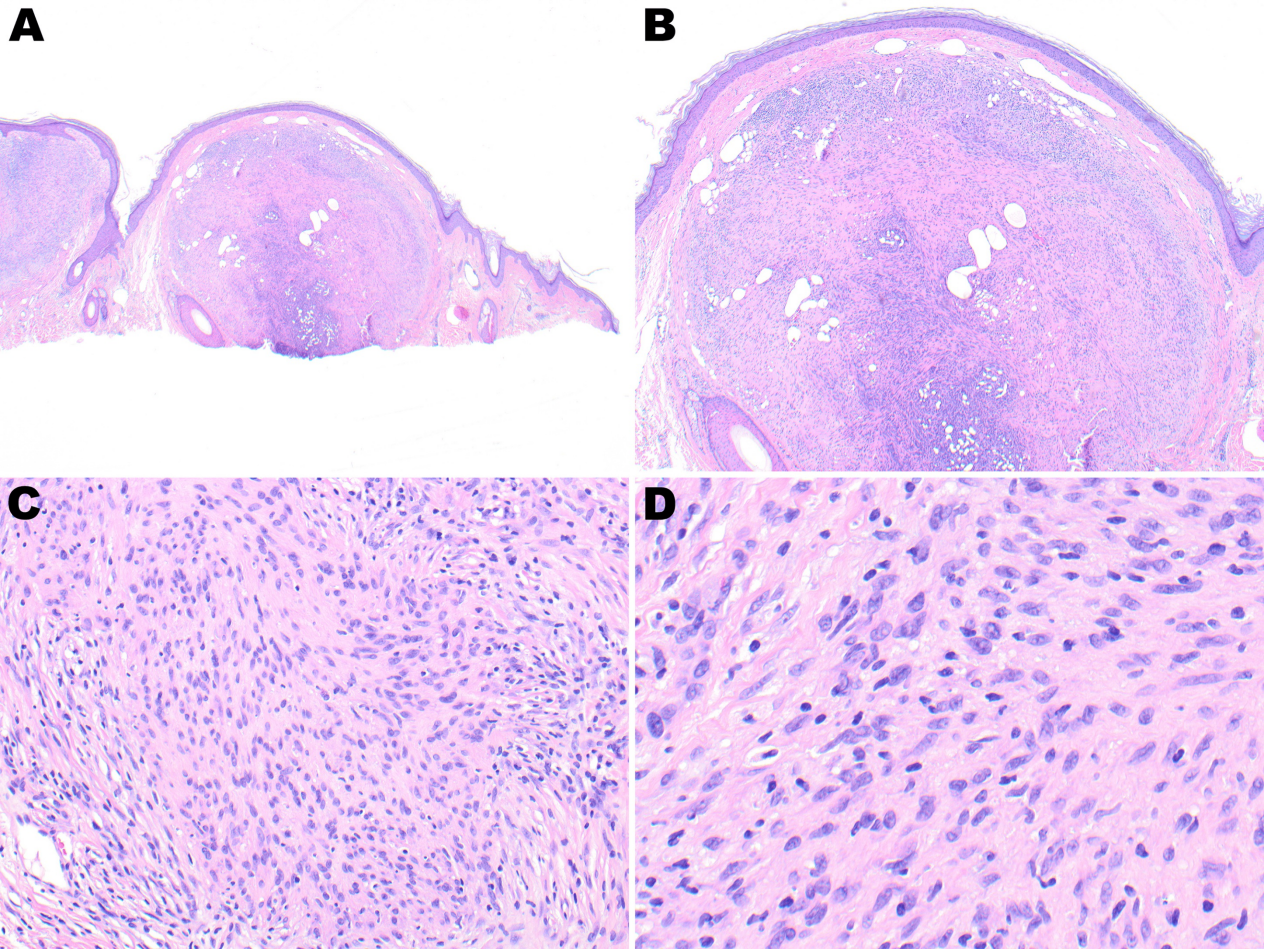
Well-circumscribed dermal-based lesion with no relation to the overlying epidermis (A, 2×; B, 4×). Mixed epithelioid and spindle cell population with mild atypia and scattered focal mitotic activity (C, 20×; D, 40×).

Histologic sections demonstrated a multinodular but well-circumscribed lesion in the dermis extending to the subcutis. There was a distinct separation between the epidermis and superficial dermis (Grenz zone). The lesion comprised atypical, mildly pleomorphic epithelioid cells arranged in nodules separated by fascicles of monotonous, mildly atypical spindle cells (Figures 1A, 1B). The epithelioid cells had hyperchromatic nuclei and irregular nuclear membranes. The spindled cells had areas of vague palisading and tapered to wavy nuclei with mild hyperchromatia scant cytoplasm, and scattered rare mitotic figures (Figures 1C, 1D). A patchy lymphocytic and histocytic infiltrate was also identified in association with the main lesion.

**Figure 2:**
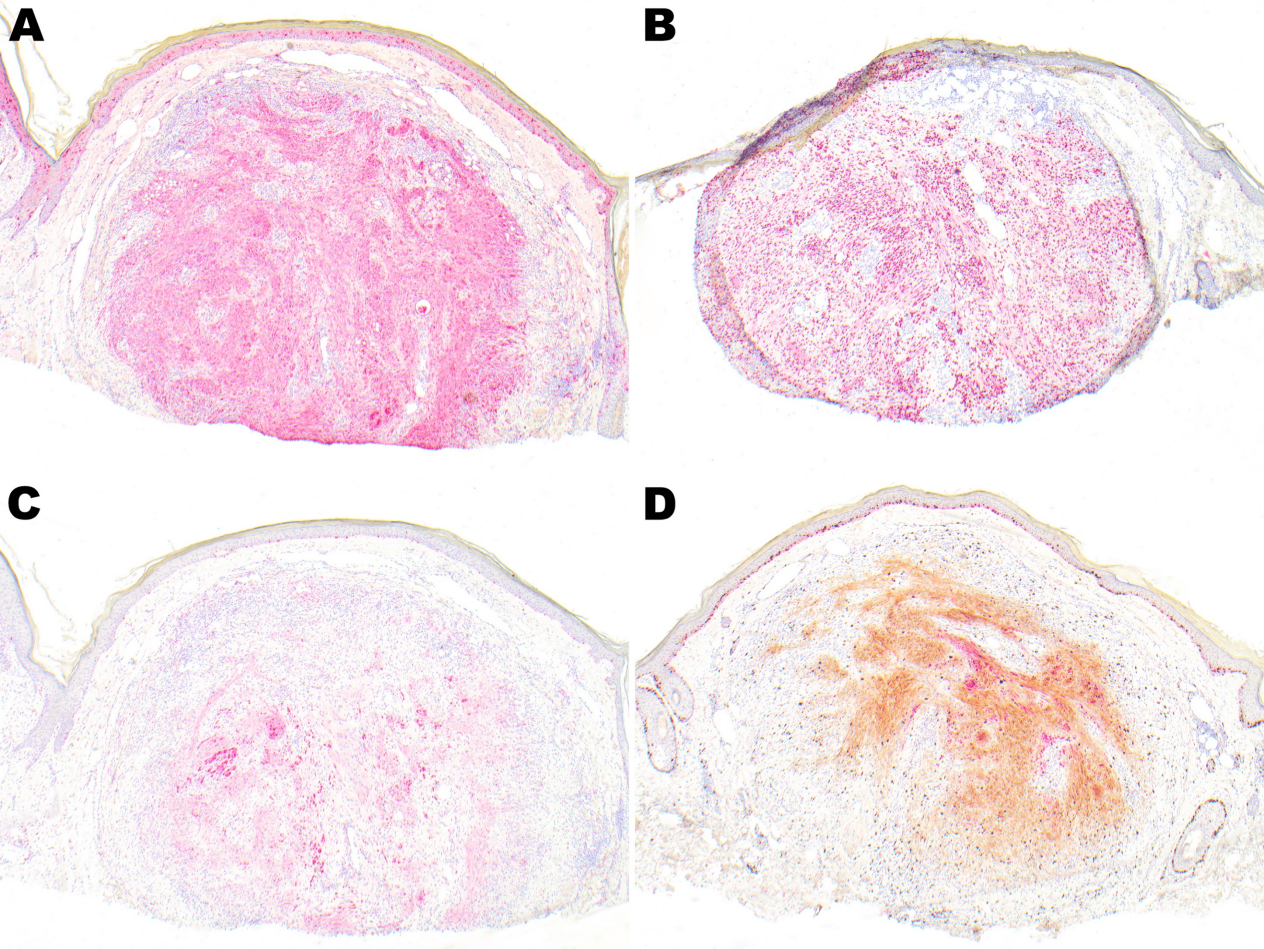
S100 stain is diffusely and strongly positive in the spindle cell population (A, 4×). SOX-10 is positive in the spindle cells and some epithelioid cells as well (B, 4×). MITF is positive in epithelioid cells and negative in spindle cells (C, 4×). Ki-67 (brown) shows the proliferative index of the tumor cells, and MART-1 (magenta) shows focal diffuse positivity in spindle cells (D, 4×). MITF - Micropthalmia Transcription Factor

**Figure 3:**
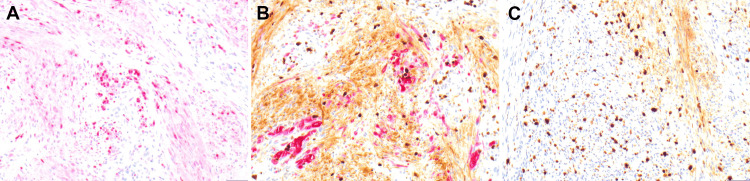
Strong expression of MITF in epithelioid cells (A, 20×). Ki-67 (brown) and MART-1 (magenta) (B, C; 20×). High power view indicating increased ki-67 index of at least 25% in the tumor cells (2B, 2C). MITF - Micropthalmia Transcription Factor

To better characterize the specimen, additional immunohistochemical stains using multiple antibodies were performed. Nuclear immunostaining for SOX10 highlighted the spindle cells, but only a subset of the epithelioid cells. S100 protein (nuclear and cytoplasmic) highlighted both spindle and epithelioid cell populations. Micropthalmia transcription factor (MITF) highlighted the epithelioid cells but was mostly negative in the spindle cells. A dual Ki-67/MART-1 immunostain revealed an increased proliferation index of at least 25% in the lesion as a whole as well as MART-1 expressing cells (Figures 2A-2D). High power view of Ki-67 in brown and MART-1 In magenta color along with expression of MITF is shown in Figures 3A-3C.

**Figure 4:**
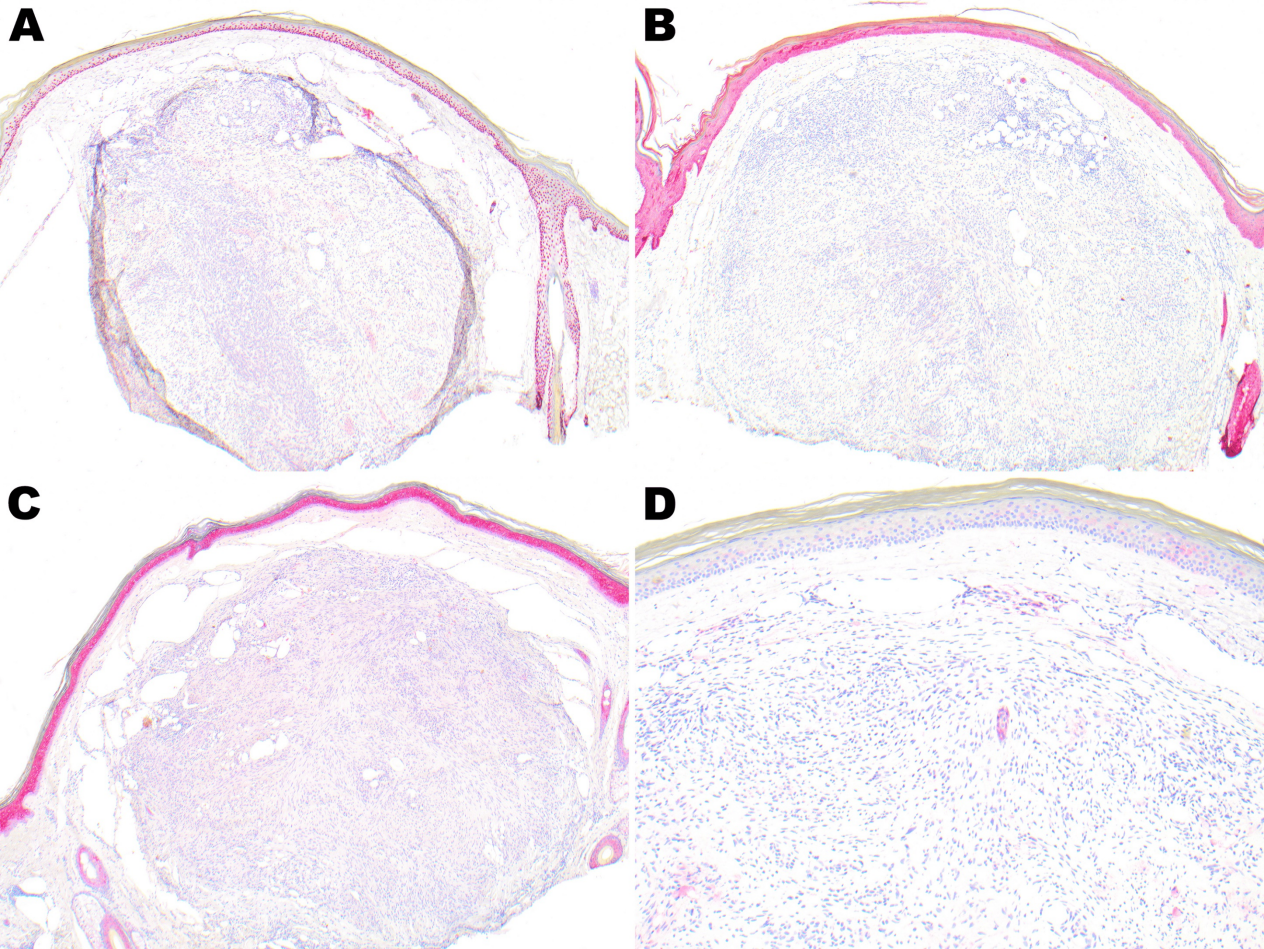
P63, AE1/AE3, and CK 5/6 stains are diffusely and strongly positive in overlying squamous epithelium (A-C, 4×). EMA shows focal, very faint staining in tumor cells (D, 10×). EMA - Epithelial Membrane Antigen

Immunostaining for p63, AE1/AE3, and CK 5/6 highlighted the surface squamous epithelium and was negative in the tumor cells. EMA showed faint positivity in a perilesional/perineurial distribution (Figures 4A-4D).

**Figure 5:**
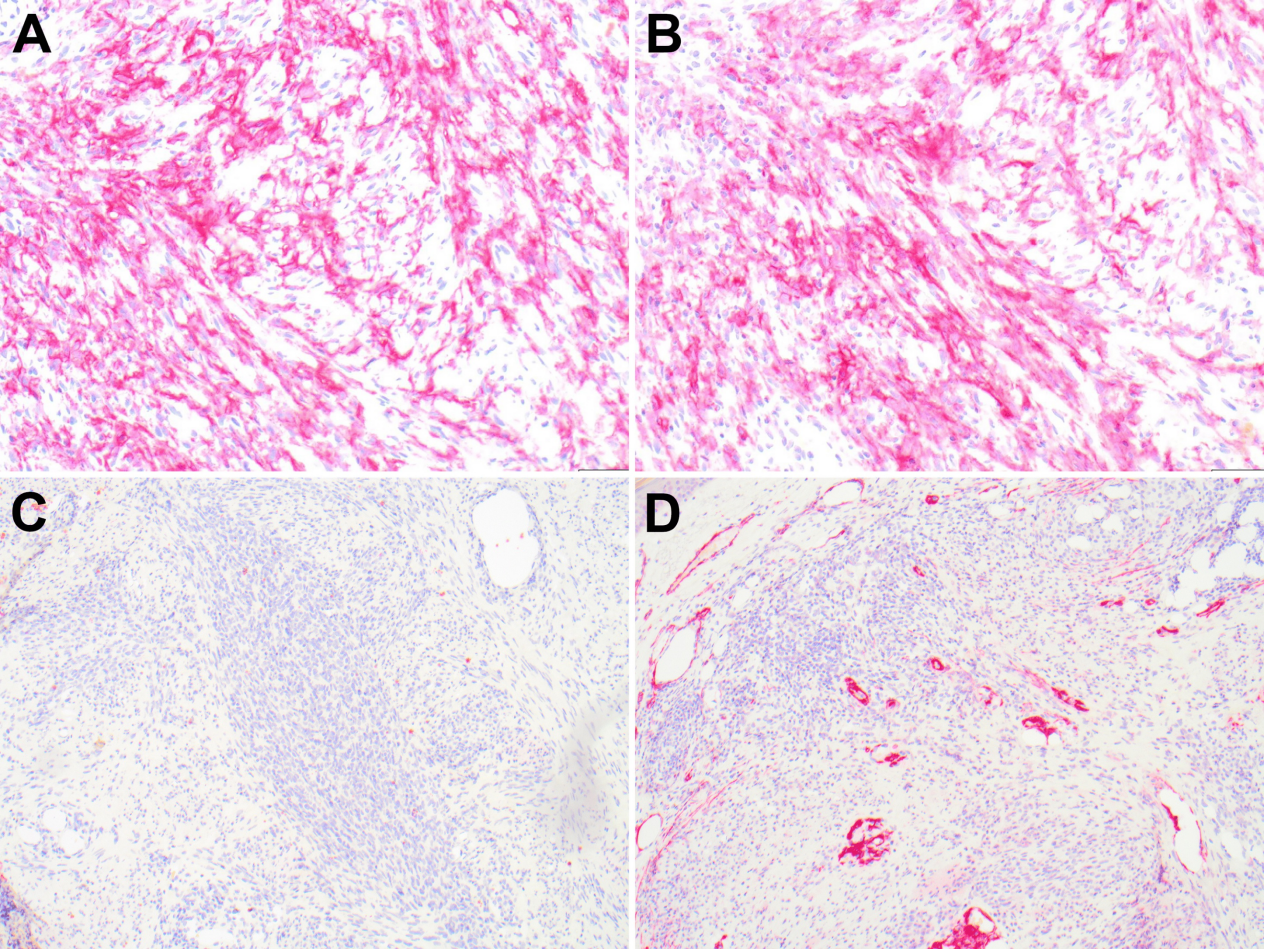
CD 34 and CD10 highlight the subset of pleomorphic and epithelioid spindle cells (A, B; 20×). Desmin and SMA are negative in tumor cells with normal expression in the blood vessels (C, D; 10×).

CD34 and CD10 showed focal positivity, highlighting the subset of pleomorphic and epithelioid spindle cells. Desmin and SMA were negative in the tumor cells and highlighted the smooth muscles of the blood vessels (Figures 5A-5D).

Based on the histopathological and immunophenotypic characteristics, a diagnosis of combined spindle cell proliferation with melanocytic and nerve sheath differentiation, consistent with neurocristic proliferation with mitotic activity but without significant atypia, was rendered. Because of the unusual features of the tumor, a complete surgical resection with negative margins was suggested. Proper post-surgical follow-up and radiological examinations were also advised to assess the deeper extent of the tumor and monitor for recurrence.

**Figure 6:**
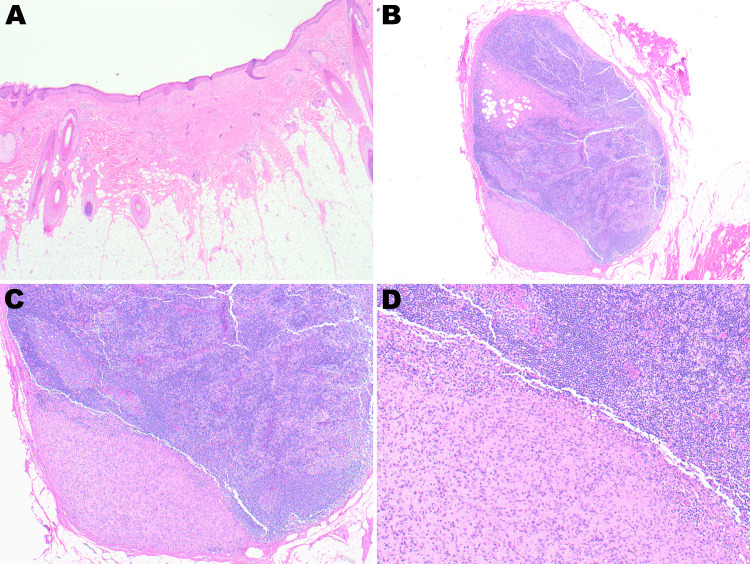
Low-power view showing an unremarkable epidermis and dermal scar (A, 10×). Medium and high-power views showing tumor metastasis to the lymph node (B-D: 10×, 20×, and 40×, respectively).

Three weeks later, a wide local excision of the tumor with sentinel lymph node dissection was performed, and the specimen was sent for histopathological examination. Microscopically, no traces of the tumor were identified in the main specimen except for the dermal scar changes at the previous biopsy site (Figure 6A). One of four lymph nodes was positive for tumor, with a deposit measuring 2.4 millimeters in the greatest dimension (Figures 6B-6E). The patient started dual immunotherapy with ipilimumab and nivolumab, but due to the intolerable side effects and toxicity, the therapy was discontinued after two cycles.

**Figure 7:**
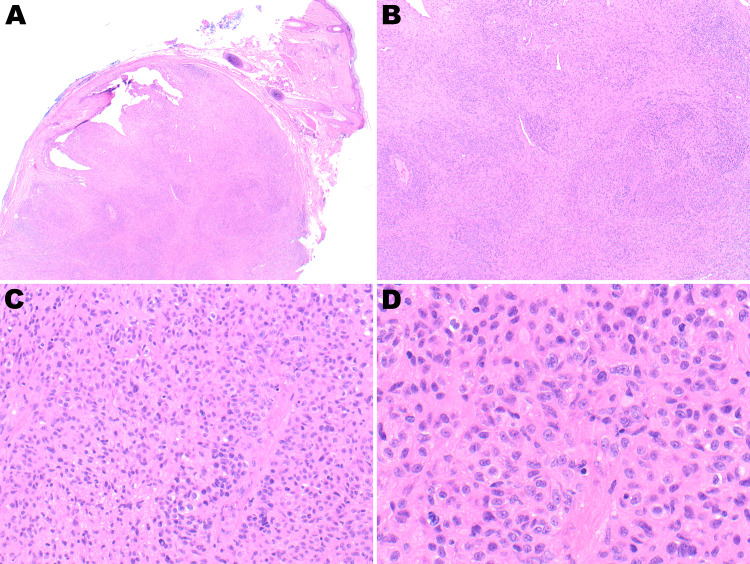
Low-power view showing the circumscribed dermal lesion with overlying epidermis (A, 4×). Medium-power view showing a hypercellular lesion composed of atypical spindle and epithelioid cells with focal mitotic activity (B-D: 10×, 20×, and 40×, respectively).

Four months later, the patient developed a satellite lesion located 5 to 7 millimeters from the original surgical resection site. A radical resection of the new satellite lesion was performed, and further histopathological evaluation demonstrated similar findings most consistent with metastatic neurocristic proliferation (Figures 7A-7D).

Comprehensive genomic profiling by next-generation sequencing (NGS) was obtained to look for targetable genetic variants. Genomic profiling revealed a high tumor mutation burden (TMB) of 27 mutations/Mega bases (Mb) and mutations in the neurofibromatosis 1 (NF1) gene and TERT promoter (Table 1). Programmed cell death Ligand 1 (PD-L1) status was evaluated by immunohistochemistry with an SP142 antibody clone, and PD-L1 expression was detected as 1+ in 2% of tumor cells.

**Table 1:** Mutated cancer-related biomarkers.

Biomarkers	Method	Analyte	Results
NF1	Seq	DNA-tumor DNA-Tumor	Pathogenic Variant Exon 5 [p.K191_R192 delinsN*] Pathogenic Variant Exon 12 [p.R440*]
TERT promoter	seq	DNA-Tumor	Pathogenic Variant c-124C >T

## Discussion

Malignant cutaneous neurocristic hamartoma (MCNH) is a rare entity, presenting diagnostic and therapeutic challenges due to its overlapping features with melanoma. Our case report describes a patient presenting with clinical and histopathological characteristics consistent with MCNH, yet exhibiting features typically associated with melanoma. This highlights the importance of comprehensive diagnostic evaluation and management strategies tailored to the individual patient [5].

The clinical presentation of MCNH can vary, but it typically presents as a solitary, elevated nodule, or plaque on the skin. The lesion might be pigmented, although this is not always the case. It can be mistaken for other skin conditions, highlighting the importance of accurate diagnosis. In our case, the patient presented with an irregular, pink nodule that had recently changed color and shape, raising concerns for potential malignancy. The most significant aspect of MCNH is its potential for high-grade malignant transformation and distant metastases. This transformation is rare, but it can happen as in our case, the patient presents with lymph node metastases of MCNH after the resection of the original tumor. Early detection and accurate diagnosis are crucial to managing this potential risk [6].

Diagnosing MCNH requires a combination of clinical assessment, histopathological examination, and immunohistochemical analysis. Clinical and histopathological differential diagnosis of MCHN may include the following conditions: Blue Nevus, characterized by spindle-shaped melanocytes in the dermis; Schwannoma, a benign tumor of Schwann cells with potentially similar histological features; Neurofibroma, which consists of a mixture of Schwann cells, fibroblasts, and mast cells; Melanoma, marked by malignant cells with atypical mitotic figures and potential for deeper invasion; Congenital Nevus, large pigmented lesions present at birth with often deeper dermal involvement; Dermal Melanocytosis, a benign proliferation of melanocytes within the dermis; Epidermal Cyst, can present as a subcutaneous nodule; and Dermatofibroma, a benign skin lesion that can be mistaken for NCH due to its nodular appearance [7].

The histological examination of our case revealed a complex architecture characterized by proliferation of neurocristic elements, consistent with MCNH. Notably, the presence of neural and melanocytic components within the lesion underscores its neuroectodermal origin. This finding aligns with previous reports describing MCNH as a neurocristic hamartoma composed of elements derived from neural crest cells [4].

Due to the potential for high-grade malignant transformation and distant metastases, surgical excision is usually recommended for MCNH lesions. Complete excision with (1 to 2 cm) clear margins is crucial to prevent recurrence or malignant progression. Regular follow-up after excision is important to monitor for any signs of recurrence or transformation. Because MCNH is so rare, there is limited research and understanding of the condition. More studies are needed to better define its clinical characteristics, underlying genetic or molecular alterations, and effective treatment approaches. This lack of information can make it challenging to manage and treat MCNH effectively [7,8]. In our case, the patient was treated with surgical excision with negative margins and immunotherapy.

Studies, such as the one conducted by Linskey et al., have contributed to our understanding of MCNH as a distinct entity separate from conventional melanoma and malignant blue nevus. These tumors exhibit unique histological features, including a biphasic pattern with neurocristic and melanocytic components, as well as an absence of BRAF mutations commonly found in conventional melanoma. This distinct molecular profile suggests a different pathogenesis for MCNH, further emphasizing the need for individualized diagnostic and therapeutic approaches [4].

In addition to its distinction from conventional melanoma, MCNH can mimic other cutaneous neoplasms, as highlighted in the study by Feinberg et al., who described a case of cutaneous neurocristic hamartoma mimicking basal cell carcinoma in a patient with xeroderma pigmentosum, emphasizing the importance of considering MCNH in the differential diagnosis of cutaneous tumors, particularly in high-risk populations [9].

Neurotropic melanoma arising from a neurocristic hamartoma represents another rare manifestation of this entity, as described in the study by Stephanie Ann Clements et al., who highlights the diverse clinical and histological spectrum of MCNH and underscores the importance of considering this entity in the differential diagnosis of neurotropic melanomas, particularly in cases exhibiting neuroectodermal differentiation [10].

Dermoscopy has emerged as a valuable tool in the evaluation of cutaneous lesions, providing insights into their morphological features and aiding in differential diagnosis. A recent study by Peter et al. described the dermoscopic features of cutaneous neurocristic hamartoma and reported its rare clinical presentation. Dermoscopic findings, including a central white scar-like area surrounded by pigmented structures, can aid in distinguishing MCNH from other melanocytic lesions, further assisting clinicians in accurate diagnosis and management [11].

Despite the typical histological features of CNH, our case exhibited concerning features resembling melanoma, including cellular atypia, increased mitotic activity, and focal areas of mild necrosis. These findings pose a diagnostic challenge, as distinguishing between MCNH and melanoma based solely on histopathology can be difficult. The coexistence of overlapping features underscores the importance of a multidisciplinary approach involving dermatologists, pathologists, and oncologists in the diagnostic workup and management of such cases [6-8].

The differential diagnosis of MCNH includes various benign and malignant entities, such as neurofibroma, schwannoma, melanocytic nevi, and melanoma. Immunohistochemical staining can aid in differentiating MCNH from its mimickers. While S100 protein and SOX10 are typically positive in both MCNH and melanoma, the absence of other melanocytic markers, such as Melan-A and HMB-45, can support a diagnosis of MCNH. Additionally, the presence of neural markers, such as S100 protein and neuron-specific enolase (NSE), further supports the diagnosis of MCNH [9,10].

Management of CNH remains challenging due to its rarity and variable clinical behavior. The primary treatment modality is surgical excision with wide margins, aiming to completely remove the lesion and minimize the risk of recurrence. However, due to the potential for malignant transformation, as demonstrated in our case, close long-term follow-up is essential to monitor for recurrence or metastasis. Adjuvant therapies, including radiotherapy, chemotherapy, and now immunotherapy or targeted therapies may be considered in cases of aggressive or recurrent disease, although their efficacy remains uncertain [8-12].

In conclusion, our case underscores the diagnostic and therapeutic challenges associated with MCNH, particularly when presenting with features overlapping with melanoma. A thorough clinical evaluation, histopathological examination, and immunohistochemical analysis are essential for accurate diagnosis and appropriate management. Further studies are warranted to elucidate the underlying pathogenesis and optimal management strategies for this rare and enigmatic entity.

## Conclusions

In summary, we have described a rare case of MCNH of the scalp with regional lymph node and satellite metastasis. Discolored scalp lesions with irregular borders would bring squamous cell carcinoma and melanoma into top differentials, but NCH should also be considered. Careful histopathological and immunohistochemical examination is important for early diagnosis and a proper treatment approach, including surveillance for the development of malignant melanoma in the future.
